# New Fossil Scorpion from the Chiapas Amber Lagerstätte

**DOI:** 10.1371/journal.pone.0133396

**Published:** 2015-08-05

**Authors:** Francisco Riquelme, Gabriel Villegas-Guzmán, Edmundo González-Santillán, Víctor Córdova-Tabares, Oscar F. Francke, Dulce Piedra-Jiménez, Emilio Estrada-Ruiz, Bibiano Luna-Castro

**Affiliations:** 1 Escuela de Estudios Superiores de Jicarero, Universidad Autónoma del Estado de Morelos, Jicarero, CP. 62909, Morelos, Mexico; 2 Laboratorio de Acarología, Escuela Nacional de Ciencias Biológicas, Instituto Politécnico Nacional, D.F., Mexico; 3 Laboratorio de Aracnología, Facultad de Ciencias, Universidad Nacional Autónoma de México, D.F, Mexico; 4 Colección Nacional de Arácnidos, Instituto de Biología, Universidad Nacional Autónoma de Mexico, D.F., Mexico; 5 Laboratorio de Ecología, Escuela Nacional de Ciencias Biológicas, Instituto Politécnico Nacional, D.F., Mexico; 6 Museo del Ámbar de Chiapas, San Cristóbal de las Casas, Chiapas, Mexico; Institute of Botany, CHINA

## Abstract

A new species of scorpion is described based on a rare entire adult male preserved in a cloudy amber from Miocene rocks in the Chiapas Highlands, south of Mexico. The amber-bearing beds in Chiapas constitute a Conservation Lagerstätte with outstanding organic preservation inside plant resin. The new species is diagnosed as having putative characters that largely correspond with the genus *Tityus* Koch, 1836 (Scorpiones, Buthidae). Accordingly, it is now referred to as *Tityus apozonalli* sp. nov. Its previously unclear phylogenetic relationship among fossil taxa of the family Buthidae from both Dominican and Mexican amber is also examined herein. Preliminarily results indicate a basal condition of *T*. *apozonalli* regarding to *Tityus geratus* Santiago-Blay and Poinar, 1988, *Tityus (Brazilotityus) hartkorni* Lourenço, 2009, and *Tityus azari* Lourenço, 2013 from Dominican amber, as was *Tityus (Brazilotityus) knodeli* Lourenço, 2014 from Mexican amber. Its close relationships with extant Neotropic *Tityus*-like subclades such as *‘Tityus clathratus’* and the subgenus *Tityus (Archaeotityus)* are also discussed. This new taxon adds to the knowledge of New World scorpions from the Miocene that are rarely found trapped in amber.

## Introduction

The fossil record of the order Scorpiones Koch, 1851 is currently comprised of 118 described species, including 21 species from amber deposits worldwide [1][2][3]. A comprehensive list of fossil scorpions has been recently given elsewhere [2]. Fossil amber scorpions have been consistently found since the Early Cretaceous. The oldest amber scorpion is *Archaeobuthus estephani* Lourenço, 2001 (family Archaeobuthidae), from the Early Cretaceous amber of Lebanon [4]. Preliminarily, *A*. *estephani* was considered a member of the superfamily Buthoidea Koch, 1837 [4]; as was *Palaeoburmesebuthus grimaldii* Lourenço, 2002 (family Incertae sedis) from mid-Cretaceous amber of Myanmar [5]. However, a phylogenetic review of the order Scorpiones has placed *A*. *estephani* and *P*. *grimaldii* outside the Buthoidea [6]. A subsequent study based on a more complete second specimen of *Palaeoburmesebuthus* suggests that it does not match with Buthoidea and extant relatives [7]. Later, the holotype of *A*. *estephani* was newly reviewed and placed in an extinct group outside the Buthoidea based on its orthobothriotaxic type F1 [8]. The later authors also presume that another primitive scorpion, *Protobuthus elegans* Lourenço and Gall, 2004 (family Protobuthidae), was initially misplaced in Buthoidea [8]. *P*. *elegans* is a non-amber fossil found in sandstones from the Early Triassic Grès à Voltzia, France. This was originally considered the earliest form associated with buthid scorpions [9]. However, Baptista and coworkers (2006) estimate that the type material of *P*. *elegans* is too fragmentary and lacks several diagnostic characters of buthid scorpions [8]. They conclude that *Protobuthus*, *Palaeoburmesebuthus* and *Archaeobuthus* are close Mesozoic relatives belonging to basal linages without extant descendants and suggest that the geological record of buthid scorpions is currently limited to the Cenozoic [8].

Fossils unequivocally belonging to the family Buthidae Koch, 1837 have been documented from Paleogene Baltic amber [2][10][11]. These have been placed into several extinct taxa: *Palaeolychas* Lourenço and Weitschat, 1996; *Palaeoprotobuthus* Lourenço and Weitschat, 2000; *Palaeoakentrobuthu*s Lourenço and Weitschat, 2000; *Palaeotityobuthus* Lourenço and Weitschat, 2000, *Palaeoananteris* Lourenço and Weitschat, 2009 (also present in contemporary Rovno amber [11]; *Palaeospinobuthus* Lourenço, Henderickx and Weitschat, 2005; *Palaeoisometrus* Lourenço and Weitschat, 2005; and the presumably misplaced species *‘Tityus’ eogenus* Menge, 1869 [12]. *Scorpio schweiggeri* Holl, 1829, the other missing specimen associated with Buthidae because of its habitus [2][10][13], however, is probably a nomen nudum [13].

In the Americas, the oldest known buthid form is a non-amber scorpion which dates back to the Paleogene; the extinct taxon *Uintascorpio halandrasorum* Perry, 1995 from the Eocene Green River Formation in western Rocky Mountains, USA [14][15]. In younger deposits of the so-called Middle America amber that comprises southern Mexico and Caribbean sites in the Neogene, several buthid scorpions have been found predominantly in Mexico and the Dominican Republic [2][3][16–22]. A few species have been assigned to the genera *Centruroides* Marx, 1890, *Microtityus* Kjellesvig-Waering, 1966 and *Tityus* Koch, 1836, such as *Centruroides beynai* Schawaller, 1979 [13], seemingly a synonym of *Centruroides nitidus* Thorell, 1876 [2]*; Tityus ambarensis* Schawaller, 1982 [16], eventually placed into *Microtityus* [17][18], currently is known as *Microtityus ambarensis* [2][16][17][18]; *Tityus geratus* Santiago-Blay and Poinar, 1988 [19]; *Tityus (Brazilotityus) hartkorni* Lourenço, 2009 [20]; and *Tityus azari* Lourenço, 2013 [21], all from the Dominican Republic. Only one species, *Tityus (Brazilotityus) knodeli* Lourenço, 2014, has been described from Chiapas, Mexico [3]. Accordingly, the genus *Tityus* is known since the Miocene in both Dominican and Chiapas amber.

The occurrence of fossil scorpions in Chiapas amber is very rare. There are only two fossils known from previously published studies [3][22]. One of them was tentatively placed within the genus *Centruroides* (status uncertain) [22], and *T*. *(Brazilotityus) knodeli* as mentioned above [3]. Scorpions from the Chiapas amber are primarily modern forms that lived in an ancient mangrove-like environment in the Miocene. Thus, there are significant intraspecific variations that allow erecting a new species closely related with the Neotropical living forms currently found in the Caribbean, Central and South America. In this study, we illustrate and diagnose a new scorpion placed into the genus *Tityus*, family Buthidae, which is now referred to as *Tityus apozonalli* nov. sp. This description is based on a single, entire, adult male specimen preserved in colorful, recrystallized, cloudy amber. The morphology of *T*. *apozonalli* is fairly close to some living *Tityus*-like species from the ‘*Tityus clathratus*’ subclade, initially included within the subgenus *Tityus (Archaeotityus)* (after Lourenço, 2006), because it shows plesiomorphic characters seen in *Tityus* scorpions [23]. It also shares some ecological traits with groups that inhabit Neotropical forests. The phylogenetic position of *T*. *apozonalli* among fossil members of the family Buthidae from the Dominican Republic and Mexican amber sites is preliminarily examined herein. As a complement, the fossil record of the family Buthidae since the Cenozoic and extinct close relatives in the Mesozoic is also briefly reviewed.

### On the fossil occurrence

**Fig 1:**
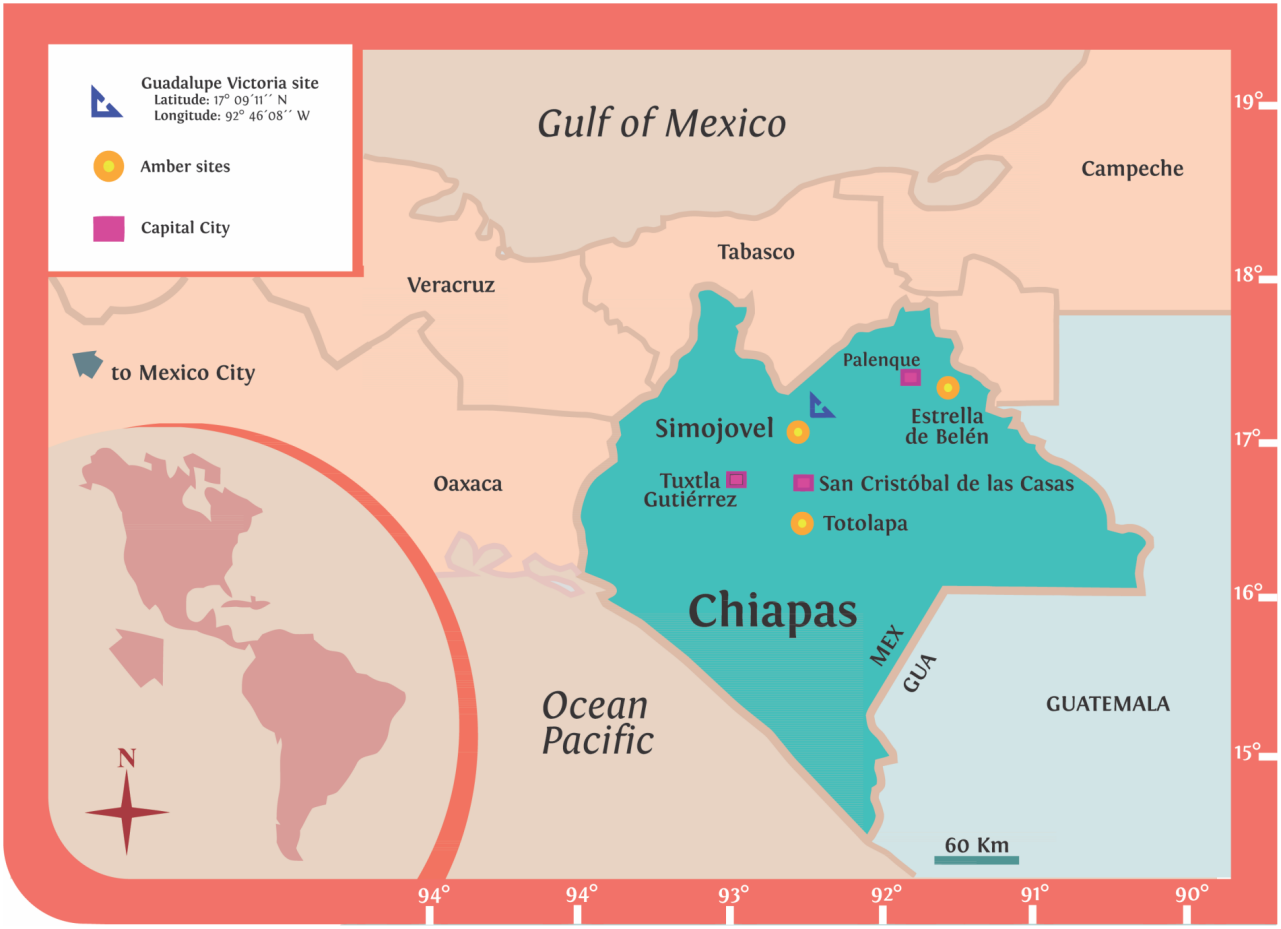
Location of the Guadalupe Victoria site near Simojovel, Chiapas, Mexico.

The fossil studied here was collected in a hand-made pit at the Guadalupe Victoria site near Simojovel, Chiapas, south of Mexico (Fig 1), which comprises two indigenous settlements: The Guadalupe Victoria I and II [24][25]. The crude amber piece was found by a native local farmer, who informed Director B. Luna of the Museo del Ámbar in San Cristobal de Las Casas, Chiapas, of his discovery. According to geographical references provided by the collector, a provenance analysis has been carried out in amber with infrared and X-ray spectroscopies. This analysis has been complemented with field geology tracking the outcrop at the amber site commonly known as the Guadalupe Victoria site [24][25]. The present amber section consists of organic-rich lignite embedded in non-fissile, coarse to fine grained sandstone with variable thickness. A ripple cross-lamination, interbedded friable shales, stratified bentonite-like clay, copious brown to red iron oxides, pyrite and other secondary clay minerals were also observed. The organic-rich lignite is the result of plant decay and soil lithogenesis. The amber section overlies a transitional-marine sequence with abundant shells, corals, calcareous debris, bioclastic limestones and consolidated sandstones. The geological setting of the Simojovel area displays rhythmic sequences that are the result of sea level fluctuations. This indicates land changes from transitional to terrestrial environments and associated climate fluctuations that span from the Oligocene-Miocene boundaries to mid-Pliocene [26]. Consequently, the lithology and sedimentary record of the amber section is consistent with a terrestrial-fluvial environment near a coastal plain [27]. Palynology in the amber strata also suggests an ancient terrestrial-mangrove environment [28].

The available published paleontological and biostratigraphic data in the Simojovel area supports that amber-bearing beds are part of the Mazantic shale and Balumtum sandstone strata with a relative age estimated in the Early to Middle Miocene, ca. 23–15 Ma. [24][25][29–32]. Another outcrop near Simojovel assigned to the La Quinta strata dated as late Oligocene has been mentioned as containing amber [29][30]. However, the location of that outcrop cannot be confirmed in current field studies [24][25]. Other author also discuss the stratigraphic position of a section of the Los Pocitos site near Simojovel–this locality also contains amber lumps- by placing it in the Simojovel Formation, which informally correlates with the La Quinta Formation in the Late Oligocene [33]. However, this age correlation is based on unpublished, unavailable biostratigraphic report apparently using Foraminifera as mentioned very briefly in [33], which increased confusion about the amber age. Geology of amber outcrops in the Mountains of Chiapas, from Totolapa to Estrella de Belén near Palenque, should be correlated with more supporting stratigraphic data in further published works.

The Guadalupe Victoria site near Simojovel along with other amber sites grouped in the Mountains of Chiapas constitute a Miocene Conservation Lagerstätte with paleobiota conspicuously preserved [24][25]. Those fossils show hard and soft tissues preservation in remarkable detail. The preservation potential has been influenced by rapid resin hardening, followed by a restriction of chemical reactions and incomplete organic decay [34]. Since the initial work by Langenheim (1966), the plant source of Chiapas amber is assigned to an extinct legume tree species of the genus *Hymenaea*, because it shares chemical signatures with extant resins of the living tree species *H*. *courbaril* and *H*. *verrucosa* [35–37]. The recent biogeochemical features and organic mineral nomenclature of Chiapas amber has also been recently reviewed [38].

The Chiapas amber also shares botanical affinities with those fossil resins occurring in the Caribbean since the Miocene, such as amber from the Dominican Republic, Haiti, Puerto Rico, Cuba, and Jamaica [30][36][39]. Recently, this amber group is known as the Middle America amber in the Neogene [24] [25]. These amber strata also have the regional tectonic setting (in the Caribbean Plate and the Central America formation) and a sedimentary regimen in common with the Simojovel sites at the Miocene [40]. However, the age correlation of amber deposits exposed in Chiapas and the Caribbean group should be interpreted with caution, because there are significant differences in depositional ages (still unstudied) that should be considered at detail.

## Material and Methods

The amber scorpion was collected in hand-made pit near the Guadalupe Victoria site, Latitude 17° 09´ 11´´ N, Longitude 92° 46´ 08´´ W, located near Simojovel, Chiapas, southern Mexico (Fig 1). The specimen is now housed at the Museo del Ámbar de Chiapas (MACH) located in San Cristóbal de Las Casas, Chiapas, Mexico. The amber collection of the MACH, including the fossil scorpion, is formally certified by the Instituto Nacional de Antropología e Historia (INAH), which protects the archeological and paleontological heritage in Mexico. No specific permits were required for the specimen description and paleontological fieldwork, which complied with all relevant regulations.

### Micrographs and measurements

Anatomical data were collected using a high-resolution microscopy with regular to adapted infrared-pass lens with LED/tungsten lamps as infrared source. A multiple image superimposition with >16 planes per image were applied for images processing and displaying [24][25][34]. Schematic drawings were hand traced using an electronic pen and a stereomicroscope, micrographs and CorelDraw X6 for graphic processing. Anatomical measurements (given in millimeters) were collected using the open source program tpsDig V. 2.17 [41]. Provenance analysis on amber was applied using infrared and X-ray spectroscopies [34][38].

### Phylogenetic analysis

The phylogenetic position of *Tityus apozonalli* sp. nov. among its fossils congeners from both Dominican and Mexican amber, Neogene, was analyzed using the TNT program [42]. Branch support values were calculated with the function tree bisection and reconnection (TBR) from existing trees, and character mapping and bootstrap values on the most parsimonious tree using a traditional heuristic search with 100,000 replicates. S1 Table shows the data matrix that includes the most informative morphological characters from the following fossil taxa: *T*. *(Brazilotityus) hartkorni* [20], *T*. *geratus* [19], *T*. *azari* [21], *T*. *(Brazilotityus) knodeli* [3], *C*. *beynai* [13], *M*. *ambarensis* [16] [17] [18], and *T*. *apozonalli*. *Palaeoananteris ukrainensi*s from Rovno amber, Paleogene [11], was used as the outgroup because of its relatively basal condition and comparable plesiomorphic characters. Distribution of qualitative and quantitative characters among taxa has been listed in S1 Appendix.

### Acronyms

BLMACH: Bibiano Luna/ Museo del Ámbar de Chiapas. INAH: Instituto Nacional de Antropología e Historia. MACH: Museo del Ámbar de Chiapas.

### Terminology

The pattern of description and terminology generally follow Stahnke, 1970 [43]. The trichobothria pattern follows Vachon, 1974 [44][45]. Metasomal carinae pattern follows Soleglad and Sissom, 2001 [46]. Pedipalp chela carinae pattern follows Prendini, 2000 [47], modified by Acosta et al., 2008 [48]. Sternum type follows Soleglad and Fet, 2003 [49]. The linguistic usage of roman numbers on tergites and metasoma segments was modified; in the following text the natural numbers were used as both ordinals and adjectives.

### Nomenclature

The electronic edition of this article conforms to the requirements of the amended International Code of Zoological Nomenclature, and hence the new names contained herein are available under that Code in the electronic edition of this article. This published work and the nomenclatural acts it contains have been registered in ZooBank, the online registration system for the ICZN. The ZooBank LSIDs (Life Science Identifiers) can be resolved and the associated information viewed through any standard web browser by appending the LSID to the prefix “http://zoobank.org/”. The LSID for this publication is: urn:lisd:zoobank.org:pub:65DAA8C5-63DF-4995-BABA-992395A9747B. The electronic edition of this work was published in a journal with an ISSN, and has been archived and is available from the following digital repositories: PubMed Central, LOCKSS

## Results and Discussion

### Systematic Paleontology

Order: Scorpiones Koch, 1851
Superfamily: Buthoidea Koch, 1837
Family: Buthidae Koch, 1837
Genus: *Tityus* Koch, 1836

*Tityus apozonalli* Riquelme, Villegas *et* González sp. nov.

ZooBank LSID: urn:lsid:zoobank.org:act: 0CF5F9D0-2E2C-40DF-9D08-EB6E62769DA6

Figs 2–8.

**Fig 2:**
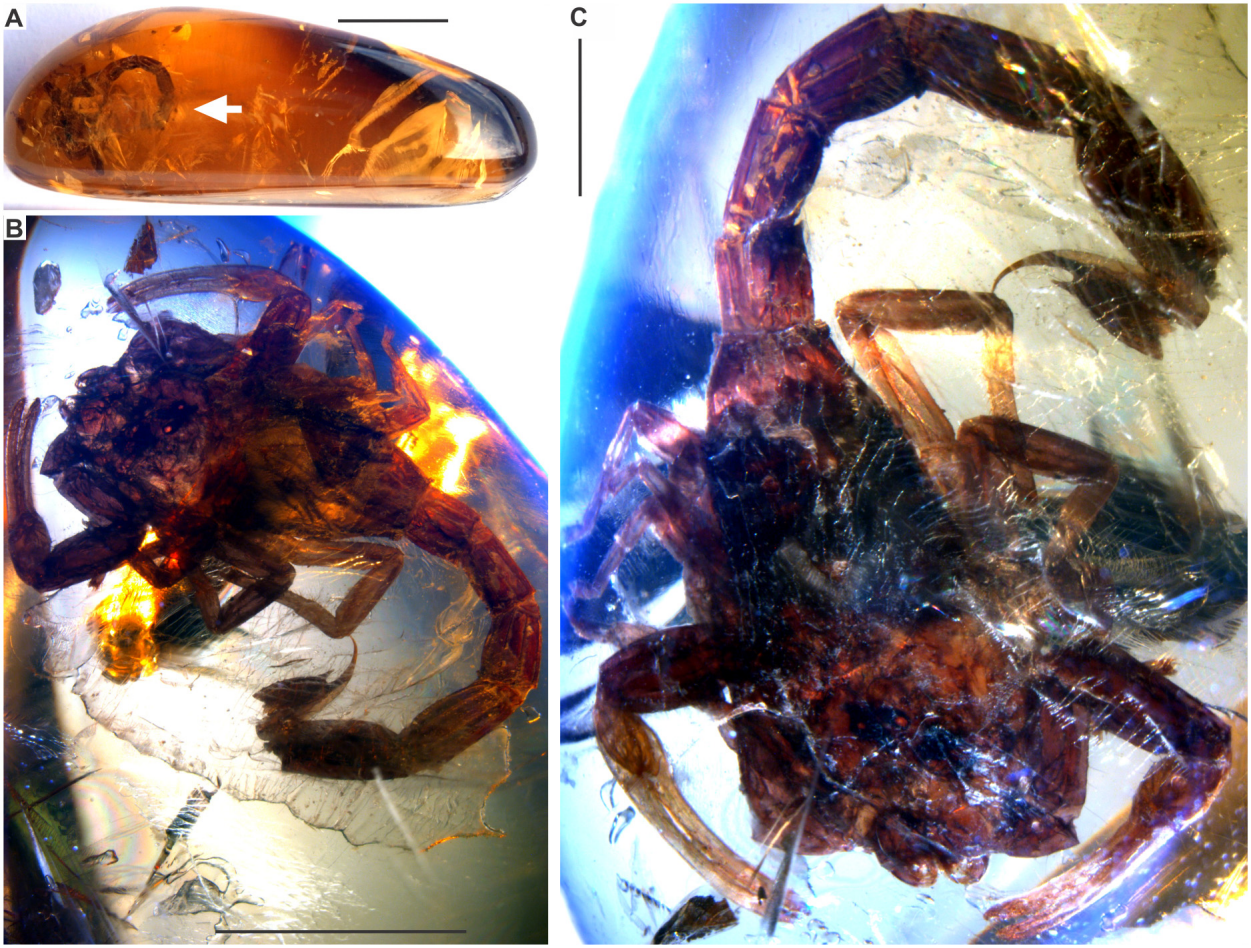
*Tityus apozonalli* sp. nov. [A]: Amber piece, arrow indicates the position of fossil inclusion, scale bar 10 mm. [B]: General view of fossil scorpion, scale bar 5 mm. [C]: Close view of the holotype male BLMACH.18, scale bar 2 mm.

**Fig 3:**
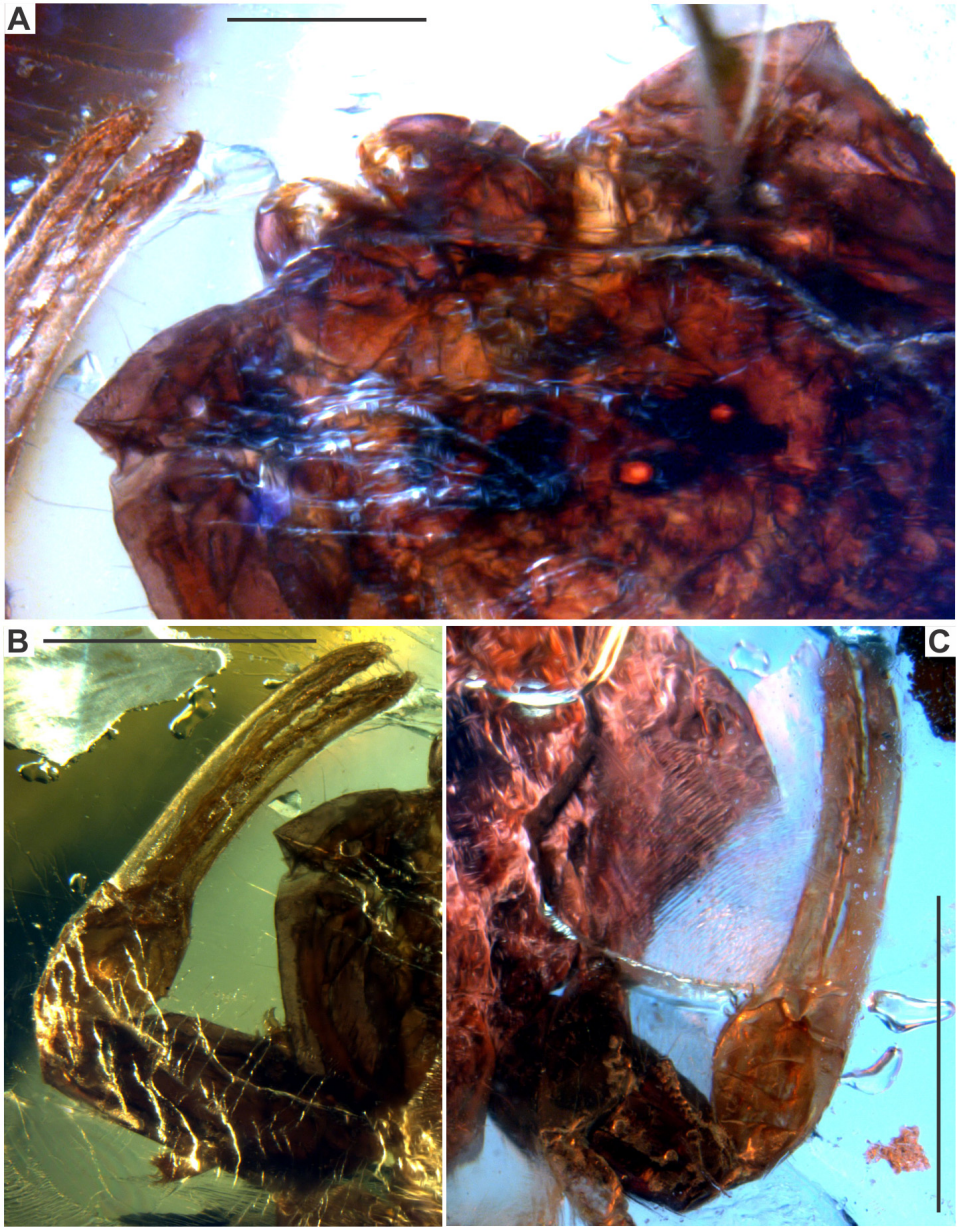
*Tityus apozonalli* sp. nov. A: Dorsal view of chelicera and partial carapace with median eyes, scale bar 1 mm. B: Left pedipalp, scale bar 2 mm. C: Right pedipalp, scale bar 2 mm.

**Fig 4:**
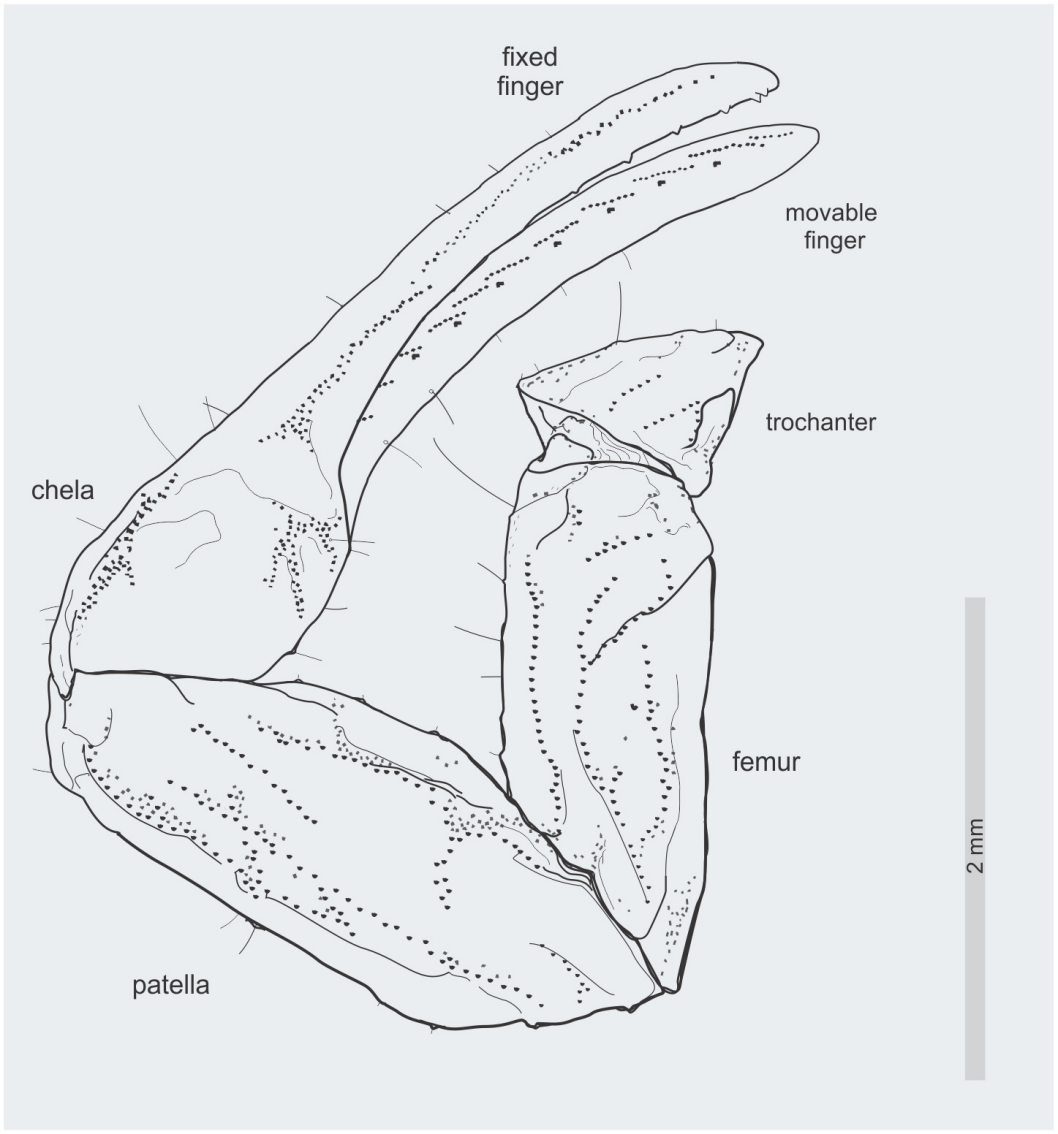
*Tityus apozonalli* sp. nov. Schematic reconstruction of left pedipalp in dorsal view, which shows discernible carinae and the pedipalp movable finger dentition.

**Fig 5:**
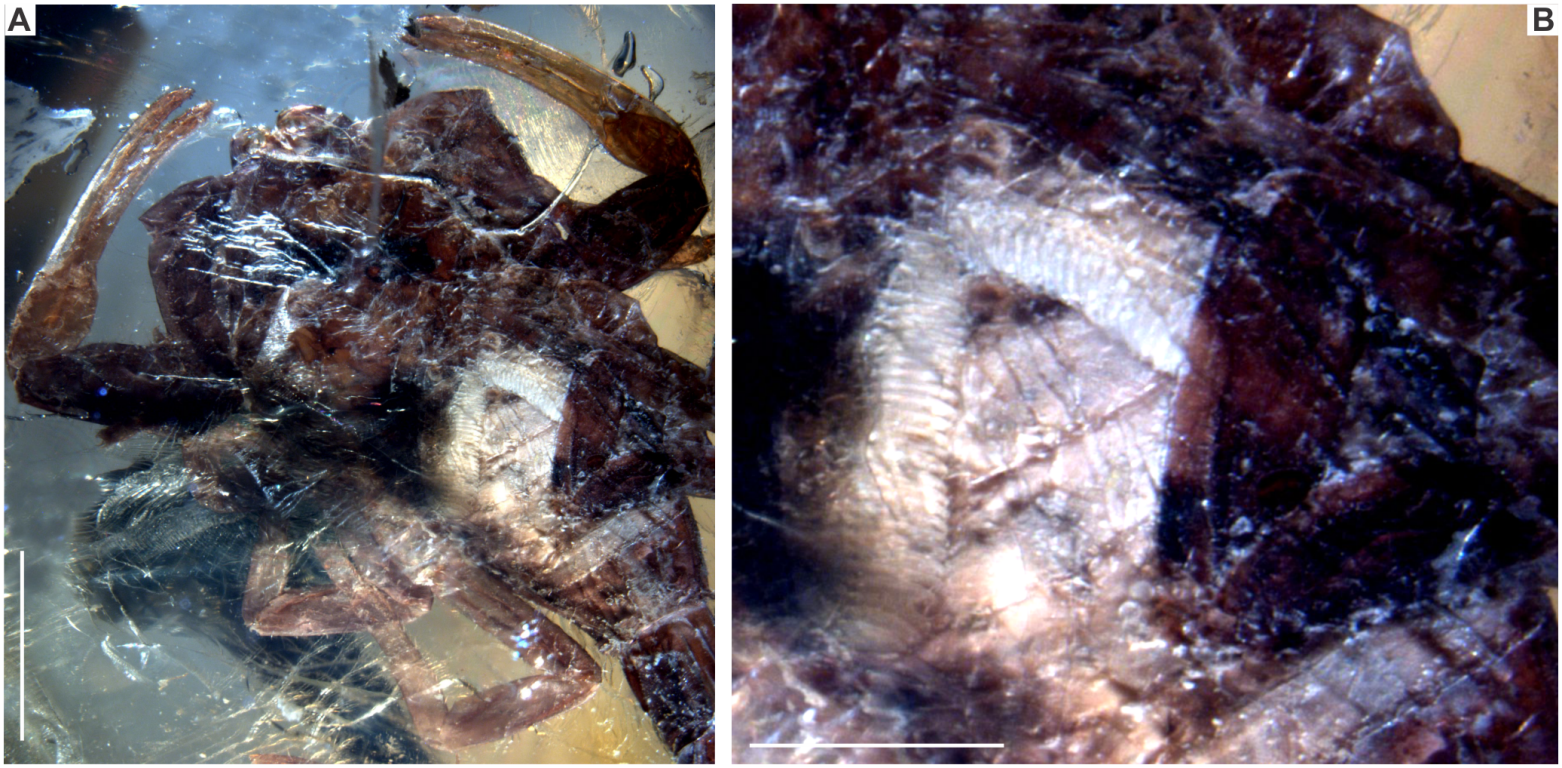
*Tityus apozonalli* sp. nov. A: general view of sternites and pectines, note the whitish color on pectines and adjacent sternites, scale bar 2 mm. B: Closer view of pectines, scale bar 1 mm.

**Fig 6:**
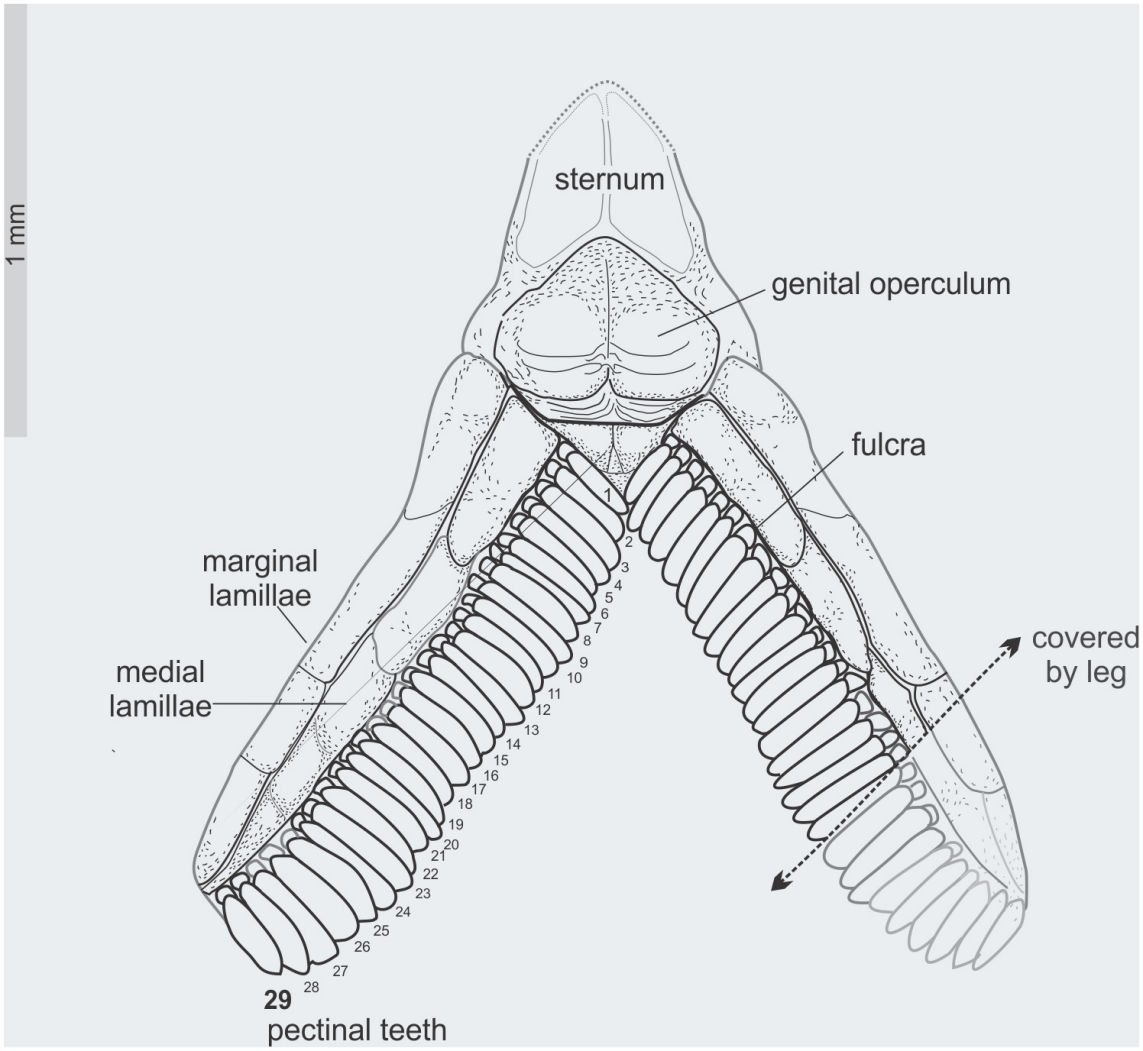
*Tityus apozonalli* sp. nov. Schematic reconstruction of sternum and pectines showing fulcra and the number of pectinal teeth, punted arrow indicate the partially visible portion as covered by leg.

**Fig 7:**
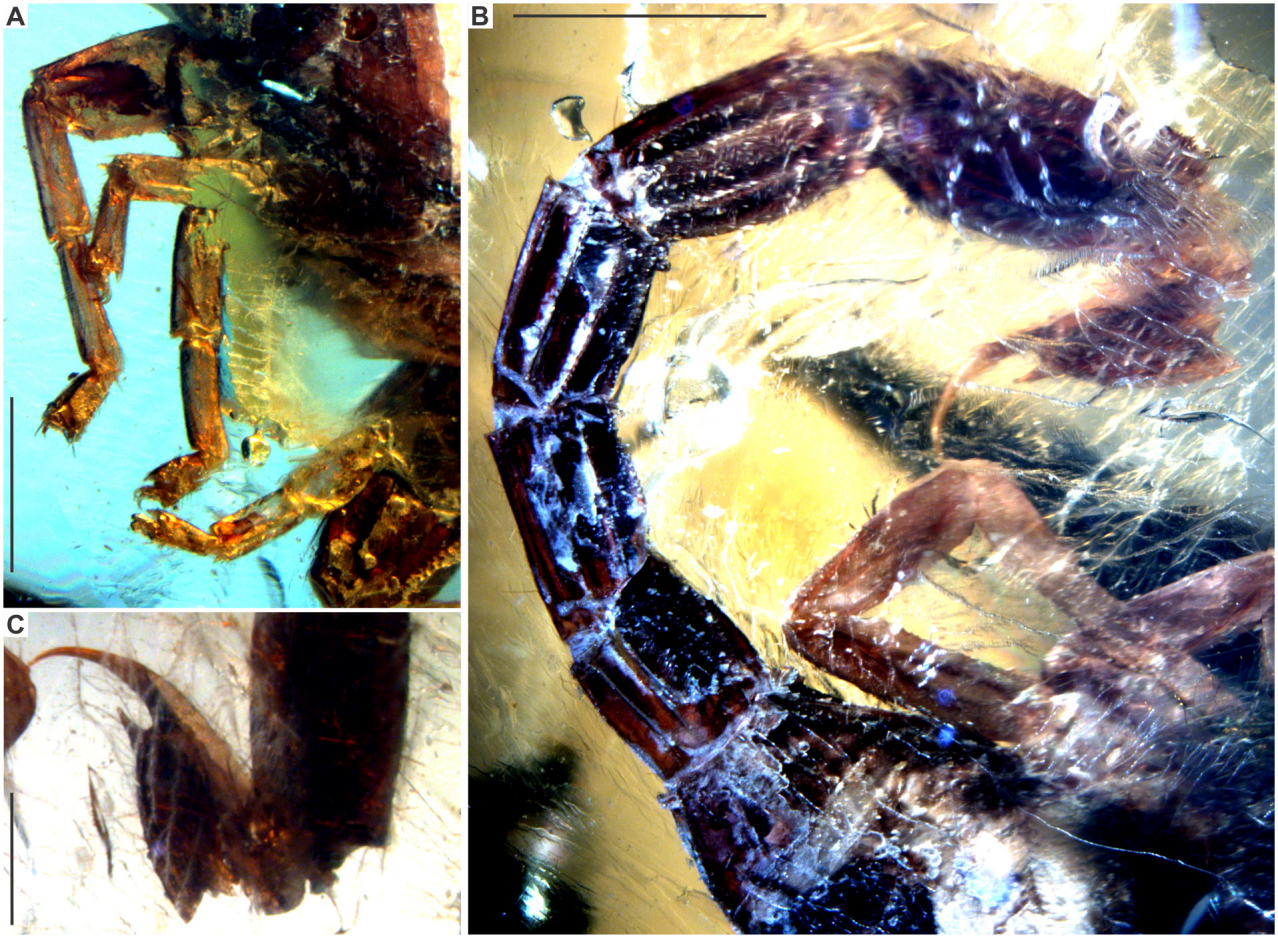
*Tityus apozonalli* sp. nov. A: Legs, scale bar 2 mm. B: Metasoma segments and telson, scale bar 2mm. C: Closer view of telson, scale bar 1 mm.

**Fig 8:**
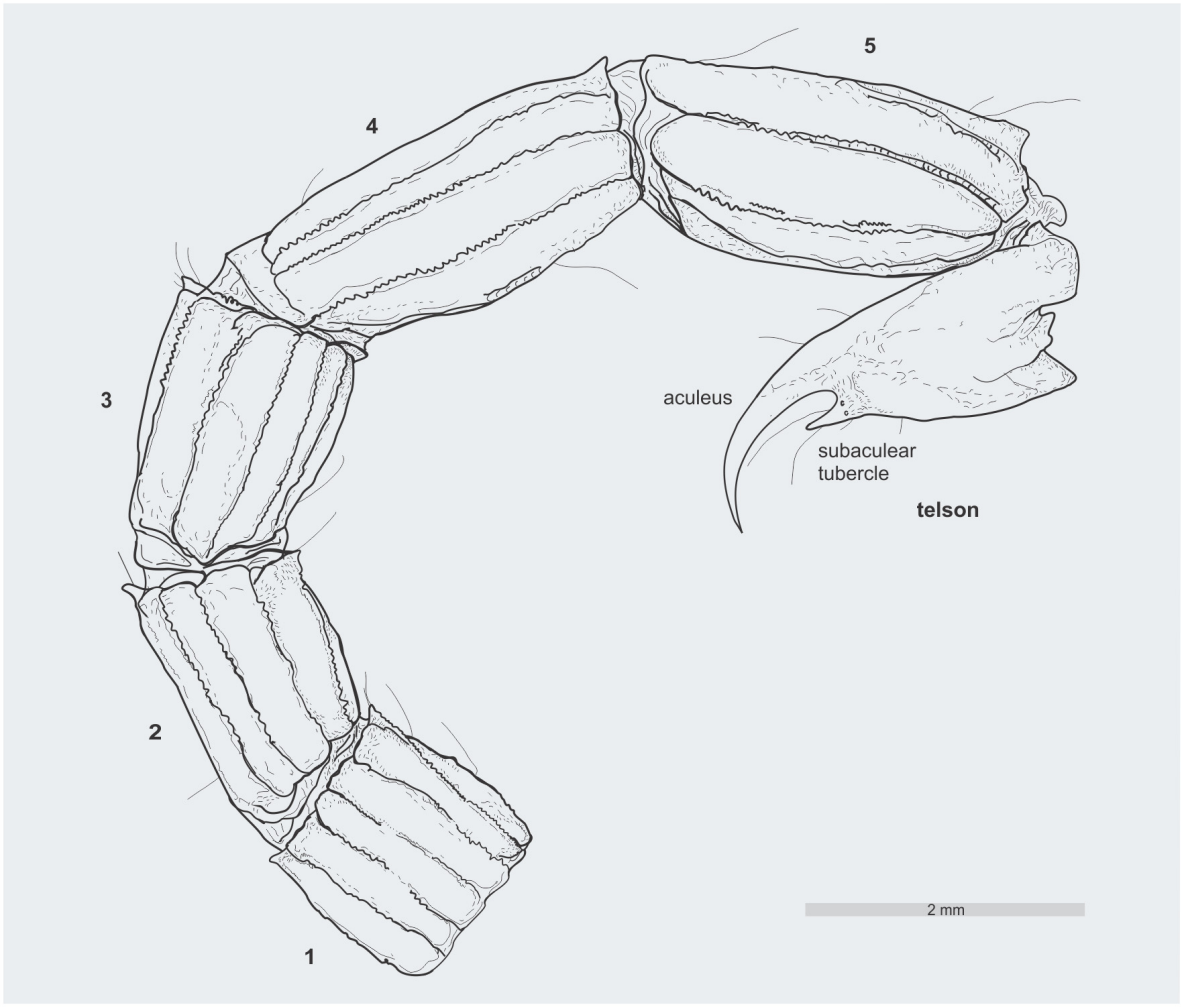
*Tityus apozonalli* sp. nov. Schematic reconstruction of the metasoma segments and telson showing aculeus and subaculear tubercle.

### Diagnosis

Small *Tityus* form, 17.8 mm in total length, reddish-brown in ground color, sparsely infuscated, pedipalp fingers + manus and legs pale yellowish. Carapace poorly emarginated anteriorly and carinae macrosculpture inconspicuous. Median eyes large. Sternum subtriangular type 1-like. Chelicerae fixed finger distinctly infuscated, with short dentition, and median denticle moderately large. Pedipalp fixed finger dentition: PD = 9 and RD = 10, MD = 9 rows of denticles; PD and RD slightly imbricated, dislocated from median rows. Movable finger PD = 11, RD = 12, MD = 10 rows of medial denticles. Trichobothrial pattern Type A, α configuration, probably orthobothriotaxic, partially visible. Pectinal tooth count 29; teeth subequal in size, relative large, fulcra present. Leg tibial spur absent, prolateral pedal spur reduced, retrolateral pedal spur moderate. Ungues short and strongly curved. Metasomal consecutive length increases progressively from segment **1** to **5**, hardly varies in width. Vesicle elongated, globose, oval-shaped, slightly granulose, with long macrosetae. Aculeus moderately large, arcuate, pale yellowish, bearing two laterodorsal macrosetae. Subaculear tubercle strong, conspicuously spiniform, lanceolate, pale yellowish, as long as one quarter of aculeus, with at least two ventrobasal granules and two visible lateral macrosetae.

### Derivation of name

“*apozonalli*”, from the Náhuatl language. This term was given to amber in the pre-Columbian times by the Aztecs, and literally means “sea bubble” or “sea foam”.

### Type material

BLMACH.18, amber inclusion, three-dimensionally preserved, entire adult male and only specimen known (Figs 2 and 3). Currently is housed at the Museo del Ámbar de Chiapas (MACH), located in San Cristóbal de Las Casas, Chiapas, Mexico.

### Locality and Horizon

The Guadalupe Victoria site, latitude 17° 07´ 58´´N, longitude 92° 48´ 19´´ W, located in the Municipality of the Simojovel de Allende, Chiapas, Mexico (Fig 1). The amber-bearing beds are part of the Mazantic shale and Balumtum sandstone strata from the Early-Middle Miocene (≈23–15 Ma).

### Taphonomic features

The specimen is embedded in golden yellow amber, tinged with orange, showing cloudy to weakly translucent glossiness, with abundant oblique microfractures (Fig 2). There is a recrystallization pattern (wave-shaped) close to the body, which is a consequence of different crystallization stages during resin hardening (Fig 2) Both fractures and recrystallization have darkened some minute anatomical elements, i.e. trichobothria, carinae and macrosculpture, which are hard to distinguish (Fig 3). There is soil, plant remains, insect parts, bubble molds and undetermined minerals embedded together with the scorpion (Fig 2). Most portions of the carapace and tergites are compressed, almost obliterated, and somewhat translucent, as a consequence of organic decay (Fig 2). This suggests that organic acids in amber have partially dissolved the cuticle. Amber is still chemically reactive in the depositional environment; this affects the organic preservation at different levels [33]. However, pedipalps, chelicerae, caudal segments and telson are less dissolved, staying tightly sclerotized (Figs 3, 5 and 7). Undetermined mineral salts are spread under sternites. These are secondary reactive products from the resin hardening. Pectines and adjacent sternites are slightly bleached (Fig 5). Legs are also translucent, flattened, with inner soft-tissues heavily degraded (Fig 7A). Telson is basally broken close to anus. The true color morphology pattern is well-preserved in general (Fig 7B and 7C).

### Color in amber preservation

Reddish-brown in ground color, somewhat translucent (Fig 2B and 2C). Carapace, chelicerae, pedipalp femur + patella reddish-brown, sparsely infuscated (Fig 3). Pedipalp fingers + manus pale yellowish. Median ocular tubercle black (Fig 3). Mesosoma segments: tergites reddish-brown, heavily infuscated (Fig 2C); sternites **3–4** whitish, others reddish-brown; pectines also whitish (Fig 5). Legs pale yellowish, with moderate brownish markings (Fig 7A). Metasomal segments and telson reddish-brown, infuscated (Fig 7B and 7C).

### Description

Holotype BLMACH.18, amber inclusion, adult male, small-sized *Tityus* morphotype, 17.8 mm in total length (Fig 2).

Prosoma: Carapace length 2.9 mm, width 3.3 mm, reddish-brown, mildly infuscated, poorly emarginated anteriorly, sublinear; carinae macrosculpture inconspicuous, most sulci and setation hardly distinguishable, anterior median notch absent. Posteromedial sulcus broad and deep. Median ocular tubercle with few minute, dusky granulates; median eyes large (≈0.23). Two lateral ocelli, one third the size of median eyes. Sternum type 1-like, almost obliterated and flattened, but in profile clearly subtriangular with median depression and paired lobes slightly visible (Fig 2B and 2C)

Chelicerae: Length 1.1 mm and width 0.7 mm, reddish-brown, almost smooth. Movable finger with internal and external distal denticles opposable, in line, about the same size. Ventral margin with a row of four smaller denticles. Dorsal margin with a moderate median denticle. Subdistal third with a row of marginal macrosetae. Serrula undiscernible. Fixed finger distinctly infuscated, with short dentition, median denticle moderately large, with a few, short, thin macrosetae ventrolaterally (Fig 3A).

Pedipalps: Total length 8.9 mm; pedipalp segments size (length / width) and (ratio): femur = (2.16/ 0.88), (r = 2.45); patella = (2.42/ 1.04), (r = 2.32); chela = (3.65 / 0.91), (r = 4.01); movable finger length = (2.55); fixed finger length = = (2.60). Femur + patella reddish-brown and sparsely infuscated. Fingers + manus pale yellowish, manus tinged with brownish striations, finger somewhat translucent. Carinae in Femur: dorsal prolateral, dorsal retrolateral, ventral prolateral and prolateral submedian with scattered irregular subserrate granules; other carinae undiscernible. On Patella: dorsal prolateral, dorsal retrolateral, ventral retrolateral, and ventral prolateral carinae finely crenulated; dorsal median costated to finely crenulated; prolateral median and prolateral ventrosubmedian finely crenulated; other carinae undiscernible. On Chela: Dorsal median carina costate; other carinae undiscernible. Pedipalp chela moderate. Fingers lacking scalloping, oblique. Pedipalp fixed finger dentition: PD = 9 and RD = 10, MD = 9 rows of denticles; PD and RD slightly imbricated, dislocated from median rows. Movable finger PD = 11, RD = 12, MD = 10 rows of medial denticles, distal row of one to three denticles missing. Trichobothrial pattern Type A-α, probably orthobothriotaxic, several trichobothria hardly distinguishable due to resin fossilization (Figs 3B, 3C and 4).

Mesosoma: Total length 4.3 mm; tergites reddish-brown, heavily infuscated, with minute wrinkles. Macrosculpture of carinae in tergites **1–6** hardly distinguishable. Carinae on tergite **7**: dorsal lateral and lateral supramedian strongly protruding and finely granular. Sternites **3–4** whitish; whereas sternites **5–7** typically reddish-brown. Sternites **3–7** ventral submedian crenulated, with coarse-to-fine granules; ventral lateral margins with carinae moderately strong, granular (Fig 2A and 2B). On sternite **7**: posterolateral carinae coarsely granular; other carinae undiscernible. Genital operculum subtriangular, longitudinally divided, male genital papillae weakly visible. Pectinal tooth count 29 (left side partially covered by leg patella **3**), average pectines: 2.61; teeth relative large, subequal in size, average tooth length: 0.40, fulcra present (Figs 5 and 6).

Legs: All segments pale yellowish, some portions hyaline and flattened, moderately setose. Dorsally granulated but basitarsi and telotarsi almost smooth. Patella strong, carinae prolateral dorsal, retrolateral dorsal and retrolateral median with moderate, subserrated granules. Setation on tarsi: retrolateral ventral with row of minor macrosetae. Tibial spur absent, prolateral pedal spur reduced, retrolateral pedal spur moderate. Ungues short and strongly curved (Fig 7A).

Metasoma: Segments moderately strong, reddish-brown and infuscated, all bearing macrosetae. Consecutive length increases progressively from segment **1** to **5**, hardly varies in width, measurements are as follows (length /width) and (ratio): Segment **1** = (1.26/1.23) (r = 1.02); **2** = (1.61/1.02) (r = 1.57); **3** = (1.64/1.15) (r = 1.42). **4** = (2.33/1.12) (r = 2.08); **5** = (2.49/1.22) (r = 2.04). Carinae on segment **1**–**5**: dorsal lateral median finely serrated, with distal spiniform projection. On segment **1**: lateral inframedian moderate, on segments **2**–**5**: strong, all serrated. On segments **1**–**5**: ventral submedian costate and strong. On segments **1–2**: ventral lateral moderate, on segments **3**–**5**: strong, all serrated. On segment **5**: ventral median moderate and finely serrated. From segment **1** to **5** carinal setation: ventral lateral counts 2:2:2:2:3, others undiscernible and/or not preserved (Figs 7B, 7C and 8).

Telson: Length 2.82 mm and width 0.96 mm, reddish-brown, sparsely infuscated. Vesicle size 1.3 length and 0.9 width, elongated, globose, oval-shaped, slightly granulose, with long macrosetae. Aculeus moderately large, arcuate, pale yellowish, with laterobasally microserration and laterodorsally bearing two visible macrosetae. Subaculear tubercle well-developed, strong, lanceolate, conspicously spiniform, sharp, pale yellowish, about 1/4 as long as aculeus, with at least two minute granules situated ventrobasally and two lateral macrosetae (Figs 7B, 7C and 8).

### Remarks

*Tityus apozonalli* sp. nov. morphologically resembles those small-sized living forms belonging to the *Tityus (Archaeotityus)* subgenus *sensu* Lourenço, 2006 that includes ‘*Tityus clathratu*s’ and ‘*Tityus columbianus*’ morphotypes currently distributed in the Caribbean Islands, Central America and northern South America [23]. It shares the presence of fulcra and strong, spiniform subaculear tubercle [23][50]. However, it considerably differs by the following diagnostic characters: (i) the morphometric values; (ii) the variation on the color and infuscation pattern; (iii) the pedipalp finger dentition that count on fixed finger PD = 9 and RD = 10, MD = 9 rows, on movable finger PD = 11, RD = 12, MD = 10 rows; (iv) the pectinal dentition that count 29; (v) the variation in the subaculear tubercle size and bearing setae. *T*. *apozonalli* also shares the presence of fulcra with others amber scorpions assigned to *Tityus* from the Dominican Republic [19–21], whereas fulcra are vestigial in *T*. *(Brazilotityus) knodeli* from Chiapas [3]. However, *T*. *apozonalli* may be primarily separated by its previously described amber fossil congeners, such as *T*. *(Brazilotityus) knodeli* [3], *T*. *geratus* [19], *T*. *(Brazilotityus) hartkorni* [20], *T*. *azari* [21], and *M*. *ambarensis* [16] [17] [18], according to the significant variation present in color/infuscation pattern, as well as the pedipalp fingers and pectinal dentition.

**Fig 9:**
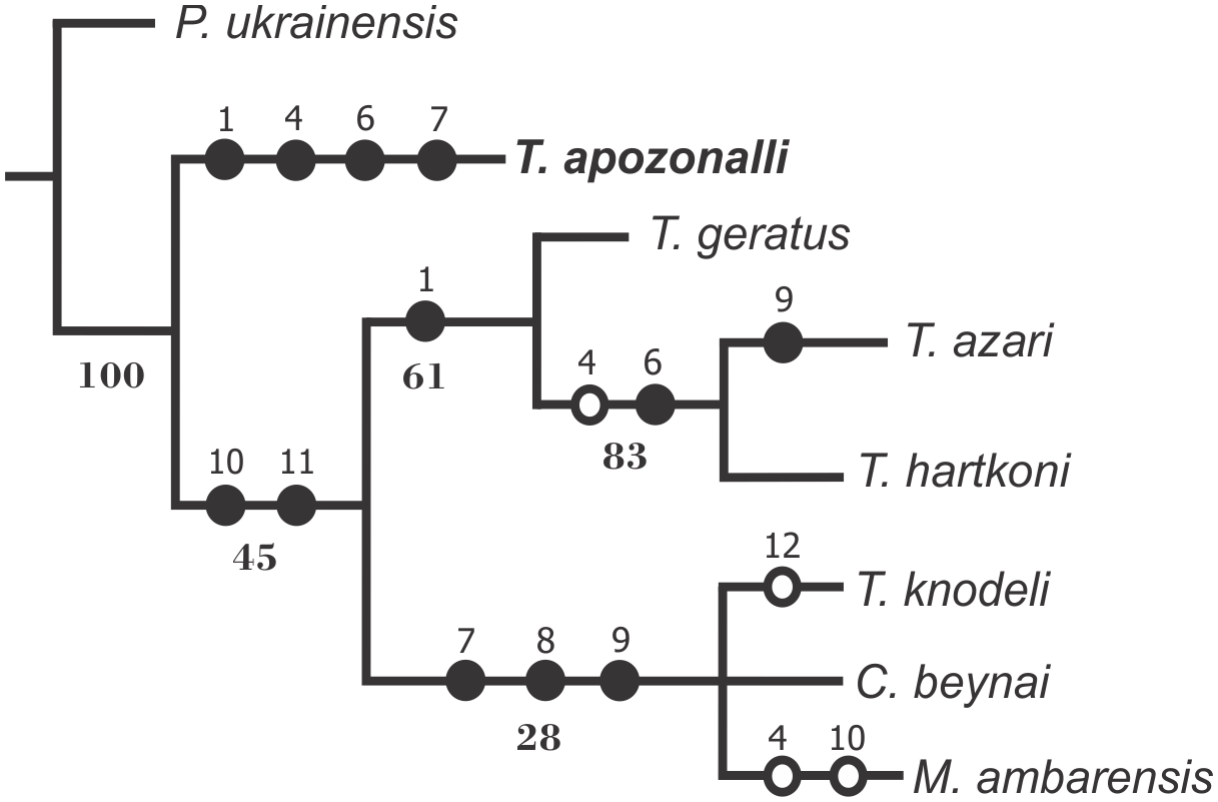
The phylogenetic status of *Tityus apozonalli* sp. nov. among fossil buthids from the Middle America amber, Neogene, inferred from data matrix in S1 Table. Characters are described in S1 Appendix. Strict consensus tree based on quantitative and qualitative characters: L = 27, CI = 0.77 and RI = 0.72. Black circles represent synapomorphies and white circle homoplasies. Numbers above circles indicate characters and numbers below branches indicate bootstrap values.

*Tityus* is a highly diverse group whose genus-level taxonomy and evolutionary relationships is currently problematic. In the past two decades, there has been important progress in identifying and separating related subclades within *Tityus* based on morphological characters, such as the subgenera proposed by Lourenço (2006) [23]. An attempt to resolve the phylogenetic relationships of *Tityus* is beyond the scope of the present paper. However, regarding the phylogenetic status of *T*. *apozonalli* among its fossil buthid congeners from both Dominican and Mexican amber, which are preliminary examined herein, the results show a single most parsimonious tree with length (L) = 27, consistency index (CI) = 0.77 and retention index (RI) = 0.72 (Fig 9). The resulting cladogram indicates a basal position of *T*. *apozonalli* which is separated from *T*. *azari* and *T*. *(Brazilotityus) hartkorni* from Dominican amber, as was *T*. *(Brazilotityus) knodeli*, previously described in Chiapas amber (Fig 9). *T*. *apozonalli* shows a plesiomorphic condition instead of *T*. *(Brazilotityus) knodeli* that seems more derived and closer to *C*. *beynai* and *M*. *ambarensis*. The plesiomorphic condition in *T*. *apozonalli* is supported consistently, but not exclusively, by the discernible fulcra that are also present in the *‘Tityus clathratus’* morphotypes, including initially within the *Tityus (Archaeotityus)* subgenera [23].

As shown in the resulting tree, the fossil members of *Tityus* seems paraphyletic, the grade of paraphyly is resolved at low resolution because of living species are not included in this analysis. However, this phylogenetic analysis provides first insights into previously unclear relationships between *Tityus* fossil members, supporting that *T*. *apozonalli* has a basal condition. Paraphyly is observed in fossil taxa with transitional states [50]. It has been discussed that fossil species with a paraphyletic behavior probably emerged by populations branching off from an ancestor who was not subsequently extinguished or as the result of adaptation by spatial differentiation, also paraphyly in fossil species might represent an extinction event [50]. Thus, the fossil diversity of *Tityus* as observed in a given time (Miocene) and space (Middle America) is discontinuous and might suggest extinction clues (Fig 9). Further phylogenetic analyses by using morphological and molecular data from living species must necessarily clarify many unknown relationships in this hugely diverse group.

**Fig 10:**
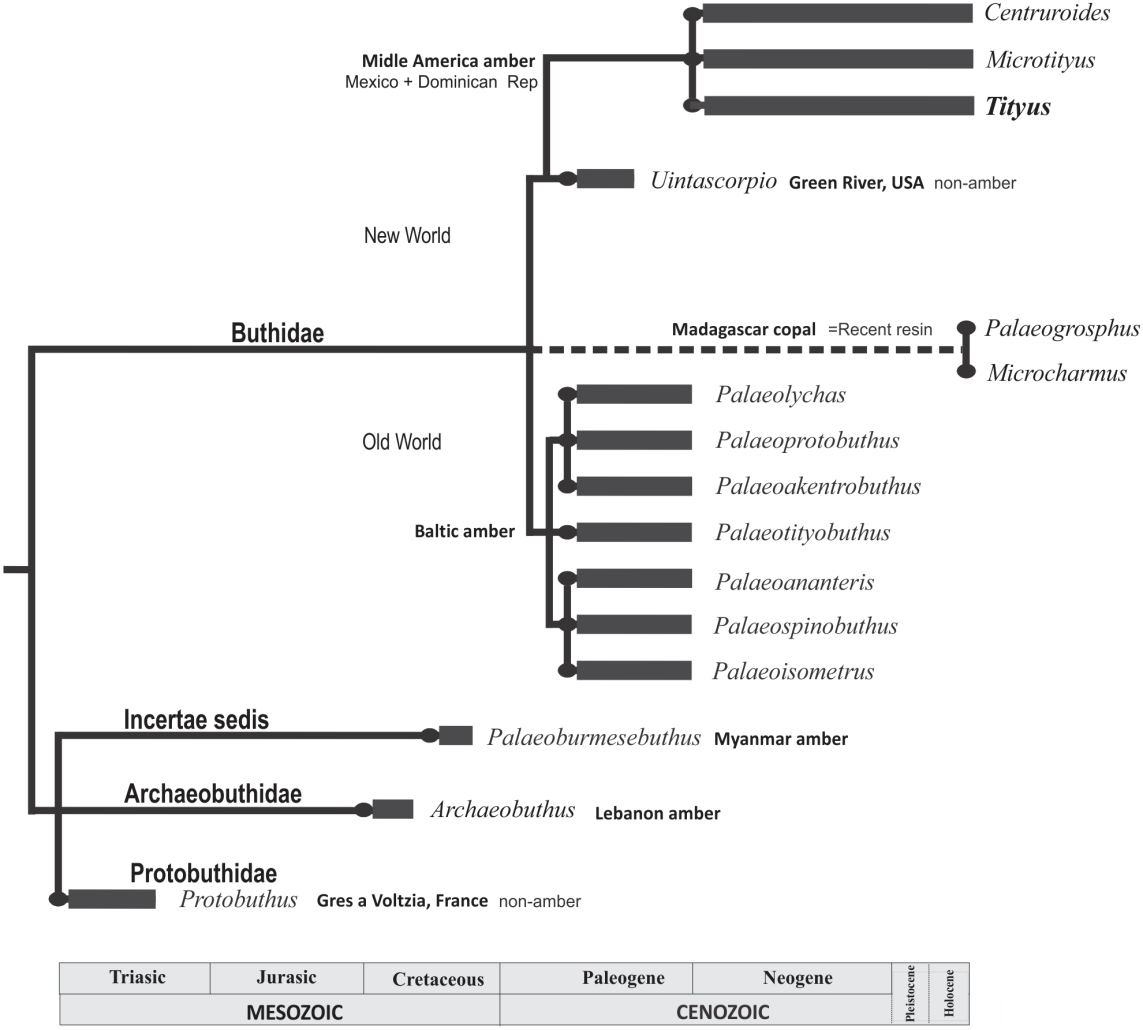
Time-scale tree that shows taxa into the family Buthidae with fossils since the Paleogene (Cenozoic) and extinct close relatives in the Mesozoic, according to the current fossil record. The extinct family Protobuthidae is here considered as a basal lineage outside the superfamily Buthoidea after Baptista *et al*. (2003) [8], and *Microcharmus* [51] is considered part of Buthidae (= Microcharmidae) after Volschenk *et al*. (2008) [52]. Note that the Copal taxa: *Palaeogrosphus copalensis*, *Palaeogrosphus jacquesi*, *Microcharmus henderickxi*, within the genera *Palaeogrosphus* and *Microcharmus* (Buthidae) [2][51][53][54], respectively, are now placed outside the geological record due to their recent depositional age.

The extant forms of the *‘Tityus clathratus’* subclade close to *T*. *apozonalli* currently live in the Neotropics of Central and northern South America [51], whereas the distribution of *T*. *apozonalli* is limited to the Chiapas amber in the southernmost part of North America during the Miocene. The current distribution of the living buthid scorpions is worldwide except Antarctica [6]. As shown by the fossil record, the family Buthidae is represented by modern forms that occur since the Paleogene of Europe with several genera found in Baltic amber, as well as single genus *Uintascorpio* found in the Paleogene of North America [14][15] (Fig 10). Several published contributions suggest that extant buthid scorpions from Asia, Africa and South America have a Gondwanaland relationship [6]. Although there is a notable gap in the Mesozoic fossil record (Fig 10), it has been preliminarily considered that the buthid scorpions emerged in Gondwana (near the Permian-Triassic boundary) as predicted by the current worldwide distribution [6]. The superfamily Buthoidea with two families: Buthidae and Microcharmidae, is considered monophyletic, neither the family Archaeobuthidae nor *Palaeoburmesebuthus* (family Incertae sedis) are currently included in the Buthoidea [6]. The assignment of the Triassic family Protobuthidae into Buthoidea is still inconclusive (Fig 10). Also, the placement of the family Microcharmidae Lourenço, 1996 as separate from the Buthidae is still under debate [6][52]. Based on mesosomal anatomy other authors propose a synonymy of Microcharmidae = Buthidae [53].

On the other hand, the genus *Tityus* is typically a New World group. The fossil specimen ‘*Tityus’ eogenus* from the Baltic amber (Central to North of Europe) seems to be initially misplaced [12]. Currently the type material of ‘*Tityus’ eogenus* is missing [12]. There are also three buthid species found in the Recent Madagascar copal (less than 250 years old); one species was assigned to the genera *Microcharmus* (family Microcharmidae) and other two into *Palaeogrosphus* [2][52][54][55]. However, these may be considered as extant species, not belonging to the fossil or even subfossil record due to the recent depositional age of copal (Fig 10).

## Conclusion

*Tityus apozonalli* sp. nov. brings the number of scorpions known from the Chiapas amber to three. However, the fossil record also includes at present other four unidentified specimens-likely *Tityus* morphotypes- now kept in some private collections and several missing specimens as result of amber fossil trading. Historically, invaluable Chiapas amber fossils have been accumulated in anachronistic and pretentious collections that illegally export fossils from Mexico. Unfortunately, the type material of *T*. *(Brazilotityus) knodeli* and *Centruroides*-like specimen that were mentioned earlier fall into this ambiguous context [3][22]. Thus, the specimens revision for comparison is basically prohibited and the information unverifiable. Also the provenance of amber pieces is questionable because Mexican amber is roughly equal to Dominican, i.e. in color, glossiness, general composition, associated plant source, similar paleobiota and geological age [34–38]. Accordingly, *T*. *apozonalli* is the first fossil scorpion in the Chiapas amber with unquestionable repository and provenance, which is particularly relevant for further research.

Endemism and species richness of the scorpion fauna in Mexico is conspicuously high compared to worldwide, with 258 described species, assigned to 26 genera and 7 families, including Buthidae, Chactidae, Diplocentridae, Euscorpiidae, Iuridae, Superstitioniidae, Typhlochactidae and Vaejovidae, which are distributed in both Nearctic and Neotropical environments [56][57]. Currently, the average number of known species from Mexico is nearly *13*.*5%* of the worldwide diversity [57], but the number is growing regularly as new species are described and taxonomic categories re-evaluated. The family Buthidae in Mexico comprises different extant species of the genus *Centruroides*, which is widely distributed in the territory [57][58], another extant species from Oaxaca near Chiapas [59] that was initially assigned to the genus *Tityopsis* Armas, 1974 but recently transferred to a new genus *Chaneke* Francke, Teruel and Santibañez-López 2014 [60], and two fossil species of *Tityus* from the Chiapas amber that now includes *T*. *apozonalli* [3]. The fossil *Centruroides*-like morphotype reported earlier needs revision [22].

Notably, there are no living species of the genus *Tityus* which have been previously described in Mexico. *Tityus* essentially shows a Neotropical distribution from northern South America to Central America and the Caribbean Islands [23]. Fossil species of *Tityus* from Chiapas amber now extend the geographical range of the genus to the southernmost part of North America in the Miocene. According to Lourenço (2014), primitive *Tityus* were likely replaced by opportunistic *Centruroides* which now are prevalent in southern Mexico [3]. However, the sedimentary and tectonic evolution of southern Mexico must play an important role in the extinction and dispersal of the Chiapas amber paleobiota, including the scorpion fauna, since the beginning of the Miocene to mid-Pliocene, ca. 23–5 Ma [61]. Accordingly, the extinction and consequent dispersion to new endemic areas was probably induced by land changes [61]. Here a vicariance interpretation suggests that past geological events in Chiapas have changed the range of amber paleobiota. The current distribution of *Tityus* scorpions supports this hypothesis.

The biogeographic relationships between multiple species of *Tityus* distributed in the northern South America and the Caribbean Islands are pointed out in several contributions [23][62][63]. As noted, the presence of discernible fulcra in *T*. *apozonalli* from Chiapas as shown in other small-sized fossil *Tityus* morphotypes from the Middle America amber seems to be a relict that shared with the present-day specimens of the *Tityus (Archaeotityus)* group, which now occur living extensively from northern South America to the Caribbean Islands. This also suggests an intense radiation at least since the Neogene in the Neotropics of southernmost North America.

## Supporting Information

S1 Appendix List of characters scored for phylogenetic analysis. (DOCX)

S1 Table Data matrix. (DOCX)
