# Supplementary material for: New Fossil Scorpion from the Chiapas Amber Lagerstätte
Source: PLoS One. 2015 Aug 5;10(8):e0133396. doi: 10.1371/journal.pone.0133396 (PMC4526686; doi:10.1371/journal.pone.0133396)
Supplement: S1 Appendix — (DOCX) [file pone.0133396.s001.docx]

**S1 Appendix**

List of characters scored for phylogenetic analysis according to Stahnke, 1971 [43], except for the trichobothria pattern *sensu* Vachon, 1974 [44][45], pedipalp chela carinae *sensu* Prendini, 2000 [47], modified by Acosta *et al.*, 2008 [48]. Length values are expressed in millimeters; all measurements were collecting using the tpsDig V. 2.17 program [41].

1. Finger fixed teeth: (0) 5-12, (1) 14-15, (2)18-32.

2. Trichobothria Type A: (0) absent, (1) present.

3. Pectinal teeth: (0) 10-12, (1) 19-20, (2) 29-30.

4. Movable finger teeth: (0) 5-12, (1) 14-15, (2) 30-33.

5. Carapace length: (0) 5-12, (1) 14-15, (2) 30-33.

6. Femur length pedipalp: (0) 1.0-1.8, (1) 2.0-2.5, (2) 2.6-3.4.

7. Patella length pedipalp: (0) 1.3-1.8, (1) 2.5-2.6, (2) 3.1-4.5.

8. Chela length: (0) 2.0-2.3, (1) 3.6-3.9, (2) 4.0-5.2.

9. Movable finger length: (0) 0.8-1.5, (1) 2.5-2.7, (2) 3.0-4.9.

10. P/R pedal spurs: (0) absent, (1) present.

11. Tibial spur: (0) absent, (1) present

12. Fulcra: (0) absent, (1) vestigial, (2) present.
