# Supplementary material for: New Fossil Scorpion from the Chiapas Amber Lagerstätte
Source: PLoS One. 2015 Aug 5;10(8):e0133396. doi: 10.1371/journal.pone.0133396 (PMC4526686; doi:10.1371/journal.pone.0133396)
Supplement: S1 Table — (DOCX) [file pone.0133396.s002.docx]

**S1 Table**

Data Matrix

| Taxa | 1 | 2 | 3 | 4 | 5 | 6 | 7 | 8 | 9 | 10 | 11 | 12 |
| --- | --- | --- | --- | --- | --- | --- | --- | --- | --- | --- | --- | --- |
| *Palaeonanteris ukrainensis* | 0 | 1 | 0 | 0 | 2 | 0 | 1 | 1 | 1 | 0 | 1 | 1 |
| *Tityus (Brazilotityus)* *knodeli* | 0 | 1 | 1 | 0 | 0 | 0 | 0 | 0 | 0 | 1 | 0 | 1 |
| *Tityus azari* | 1 | 1 | 1 | 1 | 1 | 1 | 1 | 1 | 2 | 1 | 0 | 0 |
| *Tityus (Brazilotityus)* *hartkoni* | 1 | 1 | 1 | 1 | 1 | 1 | 1 | 1 | 1 | 1 | 0 | 0 |
| *Tityus apozonalli* | 2 | 1 | 2 | 2 | 2 | 2 | 2 | 1 | 1 | 0 | 1 | 2 |
| *Centruroides beynai* | 0 | 1 | 2 | ? | 0 | 0 | 0 | ? | 0 | 1 | 0 | 2 |
| *Microtytyus ambarensis* | ? | 1 | 0 | 1 | 0 | 0 | 0 | 0 | 0 | 0 | 0 | 2 |
| *Tityus geratus* | 1 | 1 | 1 | 0 | 1 | 0 | 1 | 1 | 1 | ? | ? | ? |
